# Dual antiplatelet management in the perioperative period: updated and expanded systematic review

**DOI:** 10.1186/s13643-023-02360-9

**Published:** 2023-10-14

**Authors:** Alykhan M. Premji, Mariah B. Blegen, Alyssa M. Corley, Jesus Ulloa, Marika S. Booth, Meron Begashaw, Jody Larkin, Paul Shekelle, Mark D. Girgis, Melinda Maggard-Gibbons

**Affiliations:** 1 Veterans Health Administration, Greater Los Angeles Healthcare System, 11301 Wilshire Blvd., Los Angeles, CA 90073 USA; 2 National Clinician Scholars Program, University of California, Los Angeles, 1100 Glendon Ave., Suite 900, Los Angeles, CA 90024 USA; 3 Duke University School of Medicine, DUMC Box 104002, Durham, NC 27710 USA; 4 David Geffen School of Medicine at University of California, Los Angeles, 885 Tiverton Dr., Los Angeles, CA 90095 USA; 5 Southern California Evidence-Based Practice Center, RAND Corporation, 1776 Main Street, Santa Monica, CA 90401 USA

**Keywords:** DAPT, Perioperative Management, Surgery

## Abstract

**Background:**

Antiplatelet agents are central in the management of vascular disease. The use of dual antiplatelet therapy (DAPT) for the management of thromboembolic complications must be weighed against bleeding risk in the perioperative setting. This balance is critical in patients undergoing cardiac or non-cardiac surgery. The management of patients on DAPT for any indication (including stents) is not clear and there is limited evidence to guide decision-making. This review summarizes current evidence since 2015 regarding the occurrence of major adverse events associated with continuing, suspending, or varying DAPT in the perioperative period.

**Methods:**

A research librarian searched PubMed and Cochrane from November 30, 2015 to May 17, 2022, for relevant terms regarding adult patients on DAPT for any reason undergoing surgery, with a perioperative variation in DAPT strategy. Outcomes of interest included the occurrence of major adverse cardiac events, major adverse limb events, all-cause death, major bleeding, and reoperation. We considered withdrawal or discontinuation of DAPT as stopping either aspirin or a P2Y12 inhibitor or both agents; continuation of DAPT indicates that both drugs were given in the specified timeframe.

**Results:**

Eighteen observational studies met the inclusion criteria. No RCTs were identified, and no studies were judged to be at low risk of bias. Twelve studies reported on CABG. Withholding DAPT therapy for more than 2 days was associated with less blood loss and a slight trend favoring less transfusion and surgical re-exploration. Among five observational CABG studies, there were no statistically significant differences in patient death across DAPT management strategies. Few studies reported cardiac outcomes. The remaining studies, which were about procedures other than exclusively CABG, demonstrated mixed findings with respect to DAPT strategy, bleeding, and ischemic outcomes.

**Conclusion:**

The evidence base on the benefits and risks of different perioperative DAPT strategies for patients with stents is extremely limited. The strongest signal, which was still judged as low certainty evidence, is that suspension of DAPT for greater than 2 days prior to CABG surgery is associated with less bleeding, transfusions, and re-explorations. Different DAPT strategies’ association with other outcomes of interest, such as MACE, remains uncertain.

**Systematic review registration:**

A preregistered protocol for this review can be found on the PROSPERO International Prospective Register of systematic reviews (http://www.crd.york.ac.uk/PROSPERO/; registration number: CRD42022371032).

**Supplementary Information:**

The online version contains supplementary material available at 10.1186/s13643-023-02360-9.

## Background

Antiplatelet agents are central in the management of vascular disease. Dual antiplatelet therapy (DAPT) consisting of aspirin and a P2Y12 antagonist is used in multiple clinical contexts, for example after PCI to protect against recurrent myocardial infarction and stent thrombosis, for secondary stroke prevention after cerebrovascular ischemic events, and following certain peripheral endovascular interventions [1–7]. In patients undergoing both cardiac and non-cardiac surgery, the benefits of DAPT in terms of thromboembolic prevention must be weighed against bleeding risk. An estimated 5% of patients with coronary stents may need non-cardiac surgery within 1 year and up to 25% undergo surgery within 5 years [8, 9] A significant proportion of patients who are on DAPT for a non-cardiac indication may also require cardiac surgery [10–12].

Perioperative management of DAPT for any indication (including cardiac or peripheral stents) is not clear. Current international guidelines recommend delaying elective surgery for 1 to 6 months after coronary stent placement and continuing aspirin through the perioperative period if surgery cannot be delayed and when the procedure mandates discontinuation of a P2Y12 inhibitor [11, 12]. However, there is limited evidence to guide decision-making involving urgent surgical intervention for patients with significant ischemic or bleeding risk. These situations pose a particular challenge to clinicians who must balance the consequence of delaying surgery, the hazard of periprocedural bleeding, and the risk of thrombotic events in patients with known vascular disease.

In 2016 and 2017, we produced 2 reports and journal articles on antiplatelet therapy management for patients with stents undergoing elective surgery: 1 report focused on patients with cardiac stents [13–15] and the other on patients with peripheral vascular or cerebrovascular stents [16]. Both reports concluded that insufficient evidence was available at that time to offer clear guidance for clinical practice. In the intervening years, the use of DAPT has increased along with the need for evidence to guide clinical decisions, prompting Veterans Affairs to request an updated review. Thus, this review aims to assess the evidence regarding the occurrence of major adverse events associated with continuing, suspending, or varying DAPT in the perioperative period since 2015. The population of interest was broadened to include patients on DAPT for any reason (not just stents) and among patients undergoing any major surgery (including elective, urgent, and emergent).

## Methods

This manuscript is a condensed version of a larger report prepared for the VA [17]. The aim was to perform a systematic review regarding the occurrence of major adverse events associated with continuing, suspending, or varying DAPT in the perioperative period.

The review is reported using Preferred Reporting Items for Systematic Reviews and Meta-Analyses (PRISMA) standards [18] and the larger review was registered in PROSPERO: CRD42022371032. PROSPERO is an international database of prospectively registered systematic reviews in health and social care.

### Data sources/study selection

We conducted broad searches using terms relating to *dual anti-platelet therapy* or *double anti-platelet* or *DAPT* and *general surgery* or *surgical procedures, operative*. To identify articles relevant to the key questions, a research librarian searched PubMed and Cochrane from November 30, 2015 to May 16, 2021, and Embase from January 1 2016 to May 17, 2022. These dates were chosen so they overlapped the end date of the prior searches, which was October 2017. We limited the search to published and indexed articles involving human subjects available in the English language. Study selection was based on the eligibility criteria described above. See Additional file 1: Appendix 1 for a complete search strategy.

Four team members, working independently, screened the titles of retrieved citations. For titles deemed relevant by at least one person, abstracts were then screened independently by two team members, and subsequently a full-text review was conducted in duplicate. Any disagreements were resolved through discussion. Studies were included at the full-text level if they were original research studies of any design and had relevant outcome data presented for the patients that were on preoperative DAPT comparing at least two perioperative strategies.

We used the following PICOTs criteria to guide inclusion criteria. Studies included encompassed all original research studies of any design from 2015 to the present that included adults on DAPT for any reason undergoing major (defined as an operation requiring opening one of the major body cavities) elective, urgent, or emergent surgeries. They must have continued DAPT in the perioperative period and the DAPT strategy must have been varied (including suspension, i.e., drug or timing). Outcomes of interest were major adverse cardiac events (MACE and myocardial infarction [MI], stroke, cardiovascular death), major adverse limb events (MALE), all-cause death, and major bleeding (standardized bleeding according to Thrombolysis in Myocardial Infarction [TIMI] or Bleeding Academic Research Consortium [BARC] scores, or transfusions or blood loss) and reoperation.

### Data abstraction

Data extraction was completed in duplicate. All discrepancies were resolved with full-group discussion. At the abstract stage, information on the eligibility (whether patients were on preoperative DAPT, whether there was a comparison of patients on preoperative DAPT with at least two alternative preoperative or postoperative management groups, and whether there were postoperative outcomes included), sample size, and study design were collected. Articles meeting inclusion criteria underwent a second screening, and additional information was abstracted including categorization of comparison groups for each DAPT management strategy, patient characteristics, DAPT indication, and outcomes.

### Risk of bias assessment

To assess the risk of bias in observational studies, we used the Risk of Bias in Non-randomized Studies – of Interventions (ROBINS-I) [19]. This tool requires an assessment of whether a study is at critical, serious, moderate, or low risk of bias (or no information) in 7 domains: confounding, selection bias, bias in measurement classification of interventions, bias due to deviations from intended interventions, bias due to missing data, bias in measurement of outcomes, and bias in selection of the reported result (see Additional file 1: Appendix 2 for tool and Appendix 3 for table).

### Synthesis of results

Because studies differed significantly in DAPT strategies and outcomes measured, no meta-analytic analysis was judged clinically sensible. Therefore, the synthesis is narrative, looking at different DAPT strategies, the types of surgical procedures (predominantly coronary artery bypass graft surgery [CABG]), and outcomes. In this report, we consider withdrawal or discontinuation of DAPT as stopping either aspirin or a P2Y12 inhibitor or both agents; continuation of DAPT indicates that both drugs were given in the specified timeframe. Continuous outcomes were analyzed by using the mean or median along with a measure of dispersion (standard deviation, interquartile range) to calculate the difference and 95% confidence intervals (CI) between arms, and used mean difference as the effect measure in the presentation of the results for blood loss, and transfusions. For binary outcomes, outcome counts were used to calculate risk differences and corresponding 95% CI. Risk differences were preferred as the effect measure for these presenting data because they allow for rare events and outcomes with zero events (re-exploration and perioperative death). When a study reported an eligible outcome only as an odds ratio, we converted outcome data from other studies to odds ratios (MACE). Given the variance in outcomes, studies that were not able to be compared with another were reported narratively, and in these cases, the effect measures reported in those individual studies are reported. We created figures for outcomes with 3 or more studies and included all outcomes in the Additional file 1: Appendix 4. Graphical representations of effect sizes (mean difference, risk difference, or odds ratio) and 95% CI were plotted when available or able to be estimated using counts and sample sizes using the *metafor* package in R version 4.0.2 (R Foundation for Statistical Computing, Vienna, Austria).

### Certainty of evidence

We used the criteria of the Grading of Recommendations Assessment, Development and Evaluation (GRADE) working group [20]. GRADE assesses the certainty of the evidence based on the assessment of the following domains: risk of bias, imprecision, inconsistency, indirectness, and publication bias.

## Results

### Description of the evidence

**Fig. 1:**
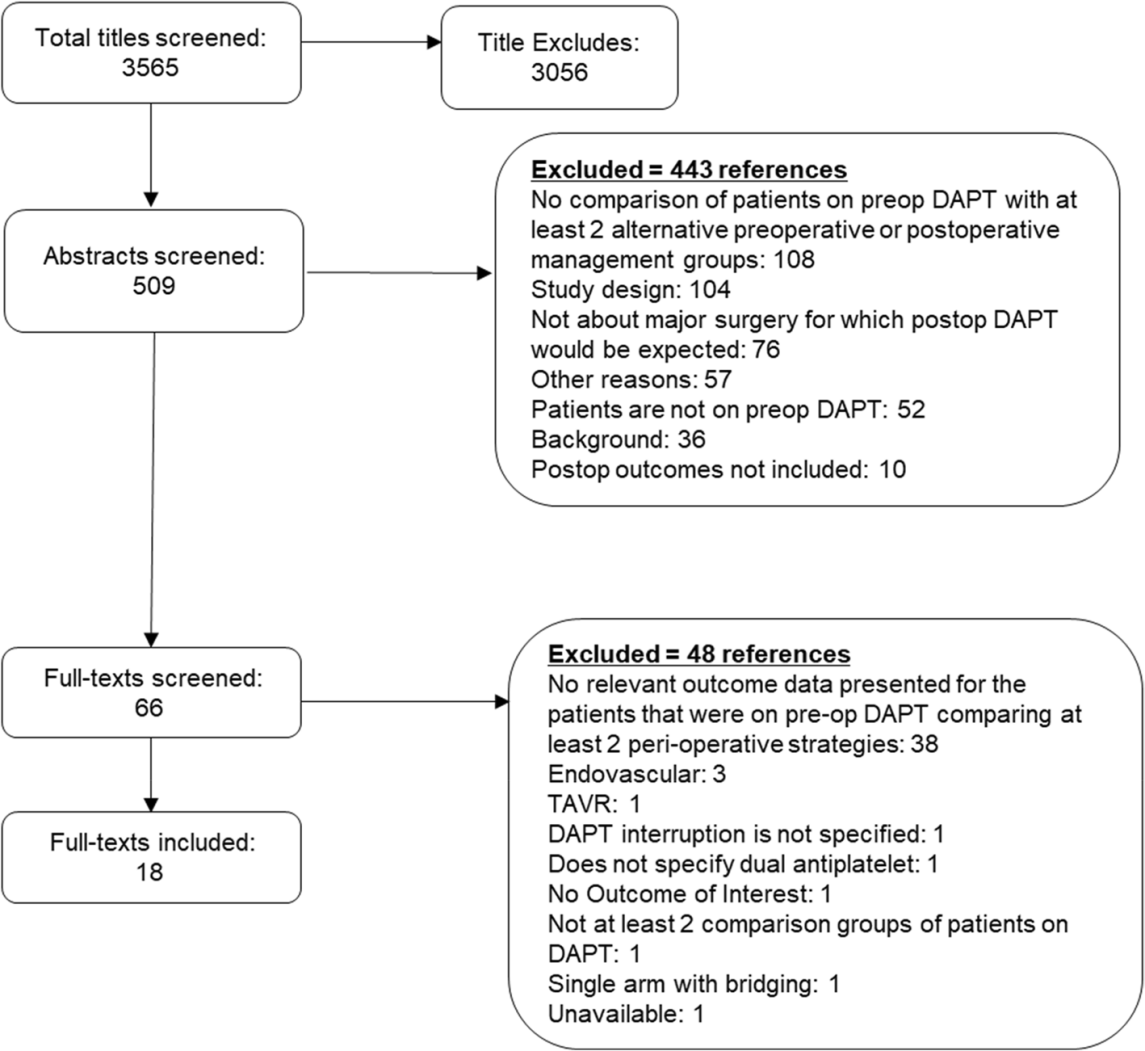
Literature flowchart

The literature search identified 3565 potentially relevant citations, 509 of which were included at the abstract screening level. From these, a total of 443 abstracts were excluded, leaving 66 publications for full-text review. See Fig. 1 for the flow and online Additional file 1: Appendix 5 for a list of excluded studies from the full-text review. Details of included publications are available in the evidence table online Additional file 1: Appendix 4.

We identified 18 publications that met the inclusion criteria, all of which were observational. Two of these [21, 22] were propensity-matched for patient characteristics such as age, sex, comorbidities, severity of surgical disease, and surgical approach. The majority of the studies were single-institution designs (*N* = 14). Most studies evaluated DAPT management at the time of CABG (*N* = 12), 3 studies evaluated surgeries categories as non-cardiac, and 1 study combined cardiac and non-cardiac surgery. Lastly, there was 1 study each evaluating hip fracture surgery and renal transplant outcomes. The strategies for perioperative management of DAPT varied: the most common approach compared different durations of time between stopping an antiplatelet agent prior to surgery (*N* = 11). Other comparisons included discontinuing 1 or both antiplatelet agents compared to continuing. One study compared a P2Y12 inhibitor discontinuation with IV tirofiban infusion (*N* = 1).

### Risk of bias

For the 18 observational studies, the quality of the studies was variable. Only 1 study was at low risk of confounding and the remainder were at medium or high risk. See online Additional file 1: Appendix 3 for the ROBINS-I assessment of studies. The studies that did not include consecutive, random, or a full sample of patients were considered at moderate risk for selection bias (*N* = 10). There was an overall low risk of bias in the classification of the interventions and deviation from these intended interventions (we judged retrospective chart review of drugs a patient received and the surgical procedure to be accurate). Missing data was not considered a significant source of bias given the use of retrospective chart reviews as the data source and the short-term (perioperative) outcomes of most studies. Finally, several studies were at moderate or high risk of measurement bias, usually due to using unvalidated or non-standard measures of bleeding(*N* = 8), such as varied methods of measuring intraoperative or postoperative blood loss. Several studies did not report cardiovascular outcomes and did provide a rationale for why clinically useful outcomes were not included. We felt that these may be at risk for reporting biases (*N* = 7).

### Patients on preoperative DAPT and undergoing CABG

#### Blood loss

**Fig. 2:**
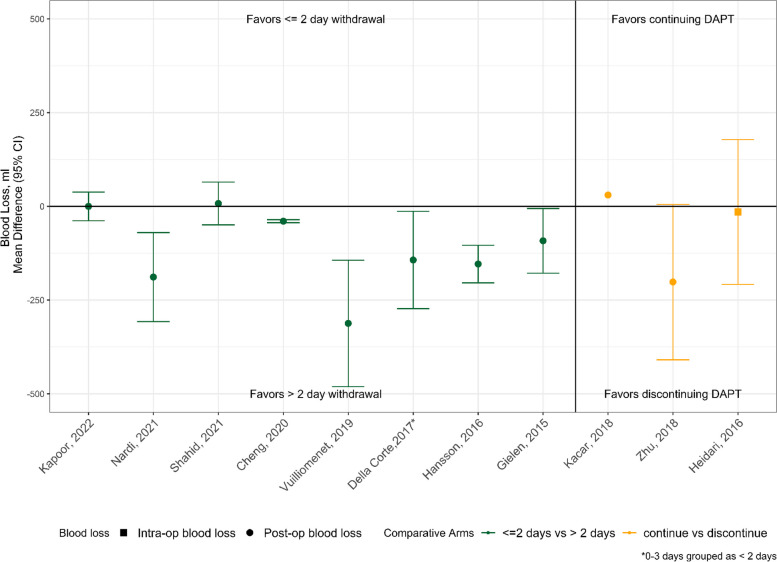
Blood loss outcomes

Eleven observational CABG studies contained sufficient data on postoperative blood loss to be presented collectively in Fig. 2. Of these, 8 compared suspending DAPT (defined as holding P2Y12 inhibition with continuation of acetylsalicylic acid [ASA]) at various preoperative timepoints, which we dichotomized as ≤ 2 days withdrawal or > 2 days withdrawal. Of note, 1 study that grouped 48–72 h was placed in the > 2 days withdrawal group [23]. A second study had comparison groups of 0–3 days and > 4 days, which were reassigned to ≤ 2 and > 2 withdrawal days, respectively [24]. The remaining 3 studies compared discontinuation DAPT, defined as stopping one or both ASA and a P2Y12 inhibitor, to continuing DAPT until surgery. In 6 of the 11 studies shown in Fig. 2, mean blood loss was statistically lower in patients that either experienced withdrawal of DAPT > 2 days preop or discontinuation of DAPT. The other 5 studies showed no significant differences in mean blood loss between DAPT management groups. Only 2 studies [25, 26] reported higher blood loss in the DAPT-withheld or discontinued groups; however, these differences were minimal (≤ 30 mL) and nonsignificant. Longer duration of suspension of DAPT therapy (i.e., for more than 2 days) favored less blood loss; however, the differences amounted to < 300 mL on average.

### Transfusions

**Fig. 3:**
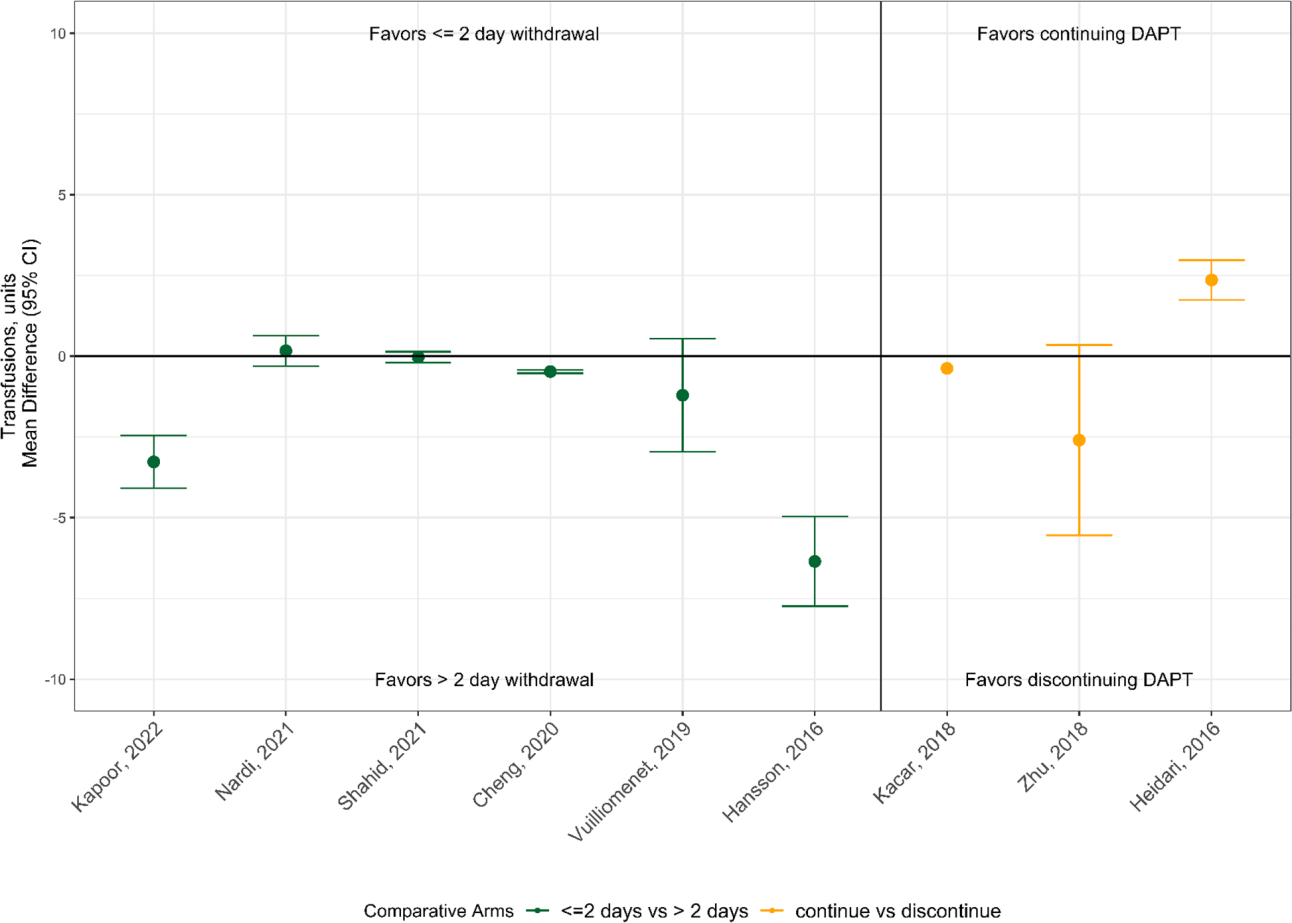
Transfusions outcomes

Differences in red blood cell transfusion requirements across DAPT strategies from the 9 observational CABG articles that reported transfusion outcomes are shown in Fig. 3. Of the 9 available studies, 4 showed less transfusion requirements for > 2 days DAPT withdrawal or discontinuing DAPT, 4 reported nonsignificant results (3 of which favored > 2 days DAPT withdrawal or discontinuation), and only 1 study [27] reported statistically more transfusions in the DAPT discontinuation group.

### Re-explorations

**Fig. 4:**
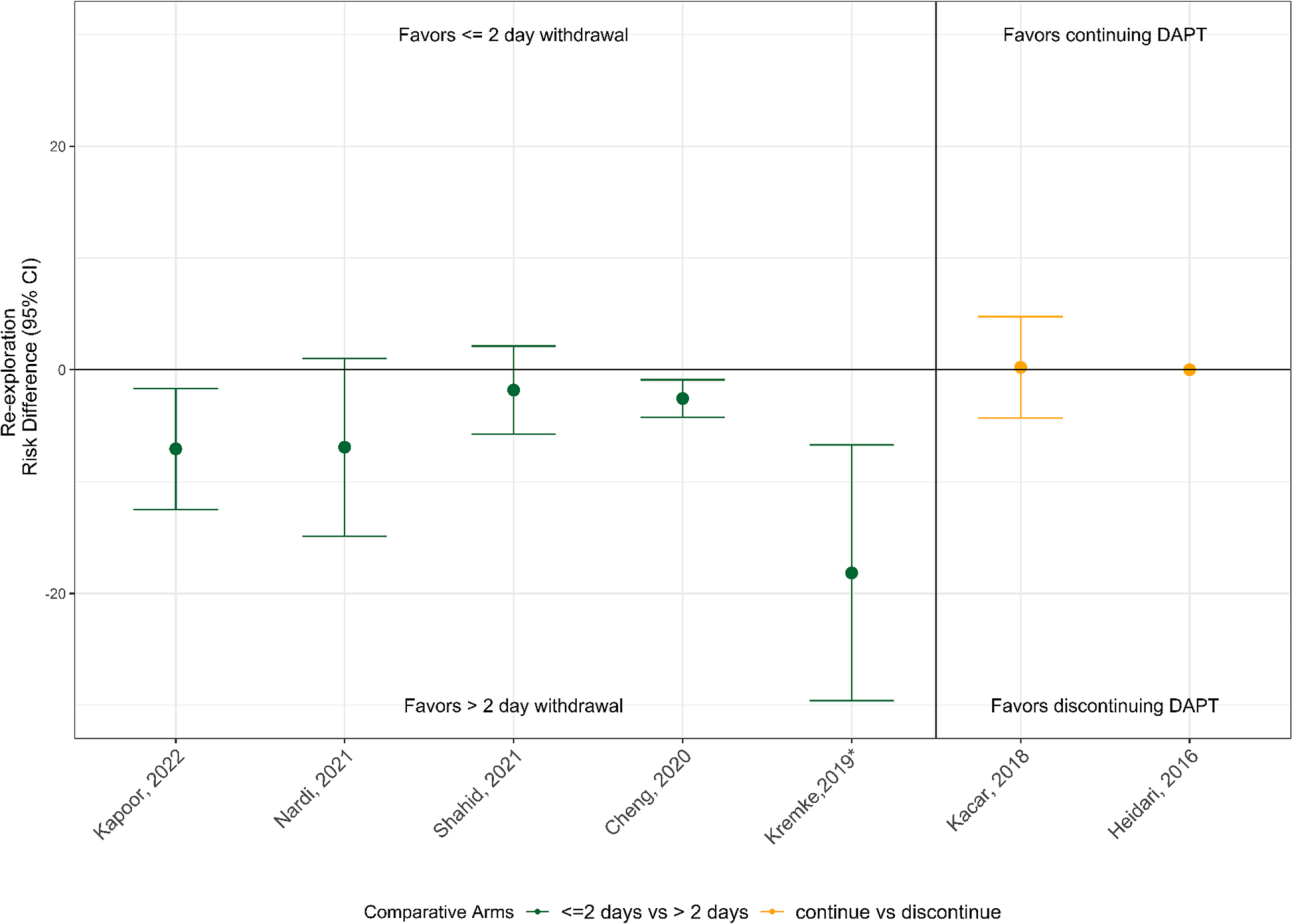
Re-exploration outcomes

Surgical re-exploration data showed a similar pattern, with all the point estimates favoring less re-exploration in patients with > 2 days DAPT withdrawal (in 2 of 5 studies this difference was not statistically significant). In contrast, the 2 studies comparing DAPT discontinuation to continuation found no difference in re-exploration (Fig. 4).

### Perioperative death

**Fig. 5:**
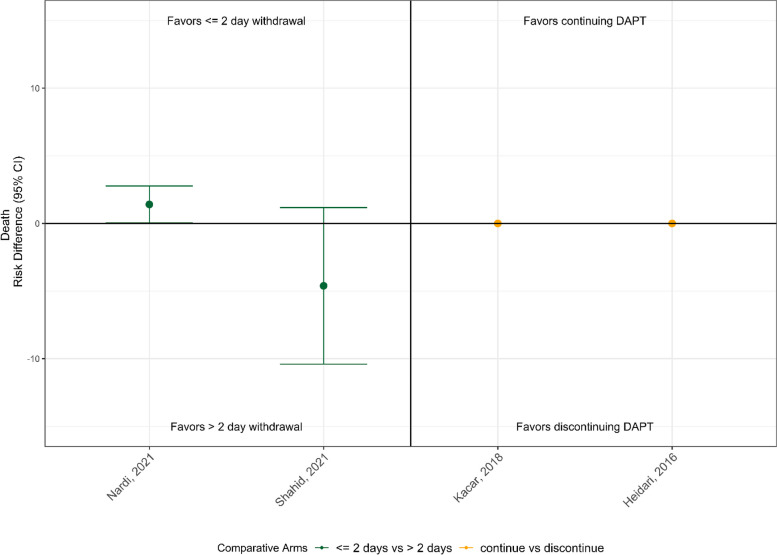
Perioperative death outcomes

There were 4 observational CABG studies that reported mortality risk differences across comparison arms (shown in Fig. 5) and 1 additional study [23] that reported mortality as odds ratios. None of these reported significant differences in patient death across DAPT management strategies.

### Cardiac outcomes

There were too few CABG studies that reported similar cardiac outcomes to the graph. Nardi and colleagues [23] observed no incidences of myocardial infarction for all DAPT management strategies, which included holding P2Y12 inhibition for 0 to 4 days prior to CABG. In a multicenter observational study of patients undergoing isolated CABG, Gielen et al. found no significant association between the last use of DAPT and MACE (odds ratio [OR] = 0.85, 95% CI [0.64, 1.14], *P* = 0.27).

### Patients on preoperative DAPT and undergoing non-cardiac surgery

Three studies reported outcomes after non-cardiac surgery [28–30]. Due to the variance in reported outcomes, it was not possible to create graphs as was done for the CABG studies, and we discuss each study narratively below.

Irie and colleagues identified 133 patients on DAPT post-cardiac stenting who underwent emergency non-cardiac surgery (57.9% abdominal, 9% vascular) and determined predictors of life-threatening and major bleeding within 180 days of surgery (*N* = 18 patients who experienced a major bleeding event) [28]. There was no significant association between the type of P2Y12 inhibitor and the risk of bleeding (unadjusted). In addition, of those who had major or life-threatening bleeding, 61% had restarted antiplatelet therapy less than 2 days after surgery compared to patients who did not develop these bleeding complications (61.1% vs 26.1%; unadjusted *P* = 0.005). After adjusting for potential confounders, overall mortality was higher in the bleeding group compared to patients without bleeding; however, the difference did not reach statistical significance (180-day mortality: 4 (22.2%) in the bleeding group vs 9 (7.8%) in no bleeding group; *P* = 0.06).

Cao and colleagues evaluated 747 patients who underwent non-cardiac surgery (33% vascular, 23% gastrointestinal surgery) within 1 year of cardiac stenting and compared outcomes among those who interrupted antiplatelet therapy and those who did not [29]. There was no association between antiplatelet therapy management and MACE after adjusting for patient factors and procedure urgency (adjusted odds ratio [aOR] = 1.23, 95% CI [0.55, 2.74], *P* = 0.62) or death within 30 days (aOR = 1.21, 95% CI [0.49, 2.98]). However, there were 83% increased odds of bleeding (defined as > 2 units transfused) among patients with no interruption of antiplatelet agent (aOR = 1.83, 95% CI [1.11, 3.01], *P* = 0.018), which the authors note tended to occur sooner after cardiac stenting.

The third study of antiplatelet management after cardiac stenting by Kim and colleagues compared discontinuing (*N* = 1750) versus continuing 1 or both antiplatelet agents (*N* = 1832) for at least 1 day prior to non-cardiac surgery across 9 institutions [30]. Here, the most common types of surgeries that antiplatelet therapy was discontinued included gynecologic, breast, head and neck, and intraabdominal surgeries, while other types such as vascular and ophthalmologic surgeries more often continued antiplatelet therapy. When comparing continuation versus discontinuation of antiplatelet therapy across all surgeries, the authors found no effect of antiplatelet discontinuation on MACE in a risk-adjusted Cox proportional hazards model (adjusted hazard ratio [HR] = 1.13, 95% CI [0.57, 2.24], *P* = 0.721) or in major bleeding when antiplatelet agents were discontinued (adjusted HR = 1.22, 95% CI [0.80, 1.87], *P* = 0.349). The authors also conclude that an optimal duration for discontinuing antiplatelet therapy is 4–8 days, as this was associated with the lowest risk of MACE (unadjusted HR = 0.12; 95% CI [0.03, 0.52], *P* = 0.019).

### Patients on preoperative DAPT and undergoing surgery for hip fracture

We identified 1 retrospective study of 122 patients taking DAPT who required fixation or hip arthroplasty for hip fracture that assessed whether the duration of DAPT discontinuation was associated with relevant clinical outcomes [21]. This study design could not isolate the effects of DAPT washout periods from confounding due to other reasons for medical delay of surgery. They found a small increased adjusted odds of 30-day mortality for each day of operative delay (OR = 1.32, 95% CI [1.03, 1.68], *P* = 0.030) but no association with units transfused among 11 patients requiring transfusion (incidence rate ratio = 1.00, 95% CI [0.87, 1.15], *P* = 0.968). The authors concluded that there was no benefit to surgical delay after hip fracture for older adults on DAPT.

### Patients on preoperative DAPT and undergoing renal transplant surgery

Our search identified 1 study that compared antiplatelet interruption before renal transplantation in 106 patients with prior coronary stent placement [31]. There were no significant differences in cardiovascular clinical outcomes, including stent thrombosis (*P* = 0.465), myocardial infarction (*P* = 0.840), MACE (*P* = 0.840), and death (*P* = 0.411), for early versus late DAPT interruption after second-generation DES or BMS placement. The authors conclude that early interruption of DAPT after stent placement in preparation for renal transplant surgery was a safe strategy and did not lead to increased ischemic complications.

### Certainty of evidence

The certainty of evidence for each of the outcomes and DAPT management strategies is shown in Table 1 below. In general, all outcomes were judged to have serious limitations due to study design and execution issues, and there were no RCTs available. All outcomes were judged to have no limitations due to directness, as the outcomes measured were judged to be both sufficiently accurately assessed and the outcomes that matter to patients. All outcomes were judged to have limitations due to imprecision, even if the directionality of results was consistent. Some outcomes were judged to have inconsistent results across studies (bleeding, transfusions, re-explorations, etc.), while some other outcomes were judged to be consistent, in part because there were so few studies (re-explorations, MACE outcomes), these latter all being judged as very low certainty evidence. In sum, there were no outcomes/DAPT strategy choices that were judged to be high or even moderate certainty of evidence. A few outcomes associated with bleeding (i.e., blood loss, transfusions) were judged to be low certainty evidence, and all other outcomes, including other possible interventions (bridging, other potential antiplatelet therapy [APT] variations) and all other outcomes (including limb outcomes), were judged to be very low certainty evidence since there was either a single observational study or no studies informing the decision.

**Table 1:** GRADE for the certainty of evidence

Outcome	Study limitations	Consistency	Directness	Precision	Certainty of evidence
Holding DAPT for more than 2 days vs less than ≤ 2 days					
*CABG surgery*					
Bleeding is less	Serious limitations	Inconsistent	Direct	Imprecise	Low
Transfusion is less	Serious limitations	Inconsistent	Direct	Imprecise	Low
Re-exploration is less	Serious limitations	Inconsistent	Direct	Imprecise	Low
Holding DAPT vs continuing DAPT					
*CABG surgery*					
No difference in bleeding	Serious limitations	Inconsistent	Direct	Imprecise	Very low
No difference in transfusions	Serious limitations	Inconsistent	Direct	Imprecise	Very low
No difference in re-exploration	Serious limitations	Consistent	Direct	Imprecise	Very low
Non-cardiac surgery					
Bleeding is less	Serious limitations	Inconsistent	Direct	Imprecise	Very low
No difference in MACE/cardiac outcomes	Serious limitations	Consistent	Direct	Imprecise	Very low

## Discussion

Perhaps the most important finding from this review is how little evidence is available to guide dual antiplatelet therapy management before surgery, despite this consequential decision being made many times, daily around the world. The studies were entirely observational in nature, with major concerns about confounding bias in patient selection amongst various DAPT strategies. The majority of included observational studies were single-center experiences, and the attempts to control for confounding were limited. Thus, our report includes no studies at low risk of bias. Furthermore, nearly all the available data are about patients with stents (mostly cardiac stents) on preoperative DAPT who are undergoing CABG. This accounted for about 75% of included studies. No studies reported limb outcomes, such as MALE. The strongest signal, in studies limited to CABG with low certainty evidence, was that the suspension of DAPT greater than 2 days was associated with less bleeding, transfusions, and re-explorations. Data about other surgical procedures, other DAPT strategies, patients with non-cardiac stents, and other outcomes were either so limited that no conclusions could be drawn or absent entirely. Although suspension of DAPT therapy for 3 days or greater was associated with less bleeding in CABG surgery, the clinical significance of this blood loss is uncertain, as the quantity of average blood loss across DAPT strategies amounted to < 300 mL of blood. We were unable to find any conclusive evidence about that strategy’s association with cardiac outcomes. Without this information, it is difficult to determine whether the risks of suspending DAPT therapy outweigh its benefits, thus practice recommendations from this evidence are limited.

In addition to the lack of studies at low risk of bias, there also was a lack of standardization in the DAPT strategies employed. There was a wide range of observed antiplatelet strategies that included holding 1 or both agents for variable amounts of time preoperatively, bridging with intravenous antiplatelet medications, or using an entirely different medication or technique to prevent excess bleeding. Additionally, there was heterogeneity in the definitions of the bleeding-related outcomes measured, with few standardized definitions, such as BARC, that were utilized. Finally, there are significant differences in the pharmacokinetic and pharmacodynamic profiles of available P2Y12 inhibitors, and any analysis may oversimplify conclusions by grouping them together.

Acknowledging these limitations, our findings pertaining to the possible benefits of holding DAPT greater than 2 days prior to CABG in terms of reduced bleeding risk are consistent with the 2021 ACC/AHA/SCAI guidelines for coronary artery revascularization and the 2017 European guidelines for dual antiplatelet therapy that recommend continuing aspirin perioperatively but holding clopidogrel for 5 days, ticagrelor for 3 days, and prasugrel for 7 days prior to elective CABG [10, 11]. In our review, we considered DAPT discontinuation or withholding as stopping 1 or both antiplatelet agents, which most often entailed holding the P2Y12 agent. Similar information is provided for non-cardiac surgery in the 2022 Chest guidelines, and the same preoperative P2Y12 withholding periods are also endorsed in current prescribing information from the major P2Y12 drug companies [32].

The best way to provide high-quality evidence on this topic would be with 1 or more well-designed RCTs. The prevalence of DAPT use among patients suggests that a study would be feasible, even in a select group of urgent general surgery patients where determining the effect of holding DAPT of varying durations would add considerable knowledge to the field. Observational studies are appealing because they are cheaper and faster to conduct than RCTs, but it is clear from the studies we found that to be more informative, observational studies will need to be more rigorous. Future observational studies should (1) include data on potential confounders to facilitate risk adjustment; (2) use large sample sizes to power subgroup analyses and multi-institutional data to reduce the impact of site and surgical team effects; (3) periodically audit the accuracy of data to ensure confidence in the dataset’s variables and values; (4) report outcomes using standardized composite endpoints such as BARC defined bleeding events and MACE. To accomplish these goals, national surgical outcome registries may consider including more granular data on the type of antiplatelets used and timing of discontinuation, as well as components of these standardized endpoints, to allow for more robust observational studies. Policymakers may also consider.

## Conclusions

The evidence base on the benefits and risks of different perioperative DAPT strategies for patients with stents is extremely limited. The strongest signal, which was still based on low certainty evidence, is that suspension of DAPT for greater than 2 days prior to CABG surgery is associated with less bleeding, transfusions, and re-explorations. Different DAPT strategies’ association with other outcomes of interest, such as MACE, remains uncertain.

### Supplementary Information

**Additional file 1:** **Appendix 1.** Search Strategies. **Appendix 2.** Risk of Bias in Non-randomised Studies – of Interventions (ROBINS-I). **Appendix 3.** Quality Assessment for Included Observational Studies. **Appendix 4.** Evidence Table. **Appendix 5.** Excluded Studies.

## Data Availability

The datasets used and/or analyzed during the current study are included in this published article and available from the corresponding author on reasonable request.

## Abbreviations

APT: Antiplatelet therapy
ASA: Acetylsalicylic acid
BARC: Bleeding Academic Research Consortium
BMS: Bare metal stent
CABG: Coronary artery bypass graft surgery
DAPT: Dual antiplatelet therapy
DES: Drug-eluting stent
GRADE: Grading of Recommendations Assessment, Development and Evaluation
MACE: Major adverse cardiac events
MALE: Major adverse limb events
MI: Myocardial infarction
PCI: Percutaneous coronary intervention
PRISMA: Preferred Reporting Items for Systematic Reviews and Meta-Analyses
ROBINS-I: Risk of Bias in Non-randomized Studies–of Interventions
TIMI: Thrombolysis in myocardial infarction
