# Supplementary material for: Dual antiplatelet management in the perioperative period: updated and expanded systematic review
Source: Syst Rev. 2023 Oct 14;12:197. doi: 10.1186/s13643-023-02360-9 (PMC10576385; doi:10.1186/s13643-023-02360-9)
Supplement: Supplementary file 1 — Additional file 1: Appendix 1. Search Strategies. Appendix 2. Risk of Bias in Non-randomised Studies – of Interventions (ROBINS-I). Appendix 3. Quality Assessment for Included Observational Studies. Appendix 4. Evidence Table. Appendix 5. Excluded Studies. [file 13643_2023_2360_MOESM1_ESM.docx]

Appendix A. Search Strategies

**PubMed
11/30/2015-5/16/22; English Language**"Dual Anti-Platelet Therapy"[Mesh] OR "dual antiplatelet*"[tiab] OR "dual anti-platelet*"[tiab] OR DAPT[tiab] OR "double antiplatelet*"[tiab] OR "double anti-platelet*"[tiab] OR (("Platelet Aggregation Inhibitors"[Mesh] OR "Factor Xa Inhibitors"[Mesh]) AND "Drug Therapy, Combination"[Mesh:NoExp])
AND
"General Surgery"[Mesh:NoExp] OR "Surgical procedures, operative"[mh] OR surgery[tiab] OR surgeries[tiab] OR surgical[tiab] OR operation[tiab] OR operations[tiab] OR amputat*[tiab] OR amputation[Mesh]

Results: 2597

**Cochrane
11/30/2015-5/16/22; English Language**[mh "Dual Anti-Platelet Therapy"] OR (([mh "Platelet Aggregation Inhibitors"] OR [mh "Factor Xa Inhibitors"]) AND [mh ^"Drug Therapy, Combination"]) OR ("dual antiplatelet*" OR "dual anti-platelet*" OR DAPT OR "double antiplatelet*" OR "double anti-platelet*"):ti,ab
AND
[mh ^"General Surgery"] OR [mh "operative surgical procedures "] OR [mh amputation] OR (surgery OR surgeries OR surgical OR operation OR operations OR amputation*):ti,ab
Results: 278

**Embase:
1/1/2016-5/17/22; English**'dual antiplatelet therapy'/exp OR ("dual antiplatelet*" OR "dual anti-platelet*" OR DAPT OR "double antiplatelet*" OR "double anti-platelet*"):ti,ab OR (('antithrombocytic agent'/exp OR 'blood clotting factor 10a inhibitor'/exp) AND 'combination drug therapy'/de)
AND
"General Surgery"/de OR 'amputation'/exp OR "operative surgical procedures"/de OR (surgery OR surgeries OR surgical OR operation OR operations OR amputat*):ti,ab
Results: 2215

Appendix B. Risk of Bias in Non-randomised Studies – of Interventions (ROBINS-I)

**Bias Domains Included in ROBINS-I**

| ***Pre-intervention*** | Risk of bias assessment is mainly distinct from assessments of randomized trials |
| --- | --- |
| **Bias due to**  **confounding** | Baseline confounding occurs when one or more prognostic variables (factors that predict the outcome of interest) also predicts the intervention received at baseline  ROBINS-I can also address time-varying confounding, which occurs when individuals switch between the interventions being compared and when post-baseline prognostic factors affect the intervention received after baseline |
| **Bias in selection of**  **participants into the**  **study** | When exclusion of some eligible participants, or the initial follow-up time of some participants, or some outcome events is related to both intervention and outcome, there will be an association between interventions and outcome even if the effects of the interventions are identical  This form of selection bias is distinct from confounding—A specific example is bias due to the inclusion of prevalent users, rather than new users, of an intervention |
| ***At intervention*** | Risk of bias assessment is mainly distinct from assessments of randomized trials |
| **Bias in classification of**  **interventions** | Bias introduced by either differential or non-differential misclassification of intervention status  Non-differential misclassification is unrelated to the outcome and will usually bias the estimated effect of intervention towards the null  Differential misclassification occurs when misclassification of intervention status is related to the outcome or the risk of the outcome, and is likely to lead to bias |
| ***Post-intervention*** | Risk of bias assessment has substantial overlap with assessments of randomized trials |
| **Bias due to deviations**  **from intended**  **interventions** | Bias that arises when there are systematic differences between experimental intervention and comparator groups in the care provided, which represent a deviation from the intended intervention(s)  Assessment of bias in this domain will depend on the type of effect of interest (either the effect of assignment to intervention or the effect of starting and adhering to intervention) |
| **Bias due to missing**  **data** | Bias that arises when later follow-up is missing for individuals initially included and followed (such as differential loss to follow-up that is affected by prognostic factors); bias due to exclusion of individuals with missing information about intervention status or other variables such as confounders |
| **Bias in measurement of**  **outcomes** | Bias introduced by either differential or non-differential errors in measurement of outcome data. Such bias can arise when outcome assessors are aware of intervention status, if different methods are used to assess outcomes in different intervention groups, or if measurement errors are related to intervention status or effects |
| **Bias in selection of the**  **reported result** | Selective reporting of results in a way that depends on the findings and prevents the estimate from being included in a meta-analysis (or other synthesis) |

Appendix C. Quality Assessment for Included Observational Studies

| **Author, Year** | **Bias Due to Confounding** | **Bias in Selection of Participants** | **Bias in Classification of Interventions** | **Bias Due to Deviations from Intended Interventions** | **Bias Due to Missing Data** | **Bias in Measurement of Outcomes** | **Bias in Selection of Reported Results** |
| --- | --- | --- | --- | --- | --- | --- | --- |
| Cao, 2022(27) | Moderate | Low | Low | Low | Low | Low | Low |
| Cheng, 2020(31) | Moderate | Low | Low | Low | Moderate | Low | Moderate |
| De Servi, 2016(32) | High | Low | Moderate | Low | Low | Low | Low |
| Della Corte, 2017(22) | Moderate | Low | Low | Moderate | Low | Low | Moderate |
| Doğan, 2017(29) | Moderate | Moderate | Low | Low | Low | Low | Low |
| Gielen, 2015(33) | Moderate | Moderate | Low | Low | Low | Moderate | Low |
| Hansson, 2016(34) | Moderate | Low | Low | Low | Low | Low | Low |
| Heidari, 2016(25) | High | High | Low | Low | Low | Moderate | Moderate |
| Irie, 2019(26) | Moderate | Moderate | Low | Low | Low | Low | Low |
| Kacar, 2018(23) | Moderate | Low | Low | Low | Moderate | High | High |
| Kapoor, 2022(35) | High | Moderate | Low | Low | Low | Moderate | Low |
| Kim, 2020(28) | Moderate | Moderate | Low | Low | Low | Low | Low |
| Kremke, 2019(20) | Low | Moderate | Low | Low | Low | Low | Moderate |
| Nardi, 2021(21) | Moderate | Moderate | Low | Low | Low | Moderate | Low |
| Shahid, 2021(24) | High | Moderate | Low | Low | Low | High | Moderate |
| Tarrant, 2020(19) | Moderate | Moderate | Low | Low | Low | Moderate | Low |
| Vuilliomenet, 2019(36) | Moderate | Low | Low | Low | Low | Moderate | Moderate |
| Zhu, 2018(37) | High | Moderate | Low | Low | Low | Moderate | Moderate |

Appendix D. Evidence Table

| **Author  Year Study design # Institutions:  Propensity (Y/N)** | **Procedure(s)** | **Pre-op DAPT Sample: n (%)**  **Comparison Groups** | **Patient Characteristics   Indication for DAPT, % Time Since Indication   Age, Years Mean (SD)  Gender [%Male or Female]** | **Bleeding Outcomes (Total or Specify)  [Report N (%), Mean (SD) or Specify]  Chest tube Drainage (cc, Total):  RBC Transfusions (Total):  Plt Transfusions (Total):  Intraop Blood Loss Volume (cc):  Reoperation:   Hematoma:   TIMI-defined Bleeding:  BARC Type:** | | | **Thrombotic/Cardiovascular Outcomes (30d or Specify) MACE: MALE: Acute MI: Stroke: Revascularization/Reintervention: Major Amputation: CV Death: All-cause Death:** | | | **Statistical Methods  Adjustment?**  **[If Y: Propensity, Multivariable Regression, other? What Were Adjustment Variables?]** | **Comments** |
| --- | --- | --- | --- | --- | --- | --- | --- | --- | --- | --- | --- |
|  |  |  |  | Continued DAPT (or DAPT held ≤ 2d) | Discontinued DAPT (or DAPT held > 2d) | Bridged / Other: | Continued DAPT (or DAPT held ≤ 2d) | Discontinued DAPT (or DAPT held > 2d) | Bridged / Other: |  |  |
| Gielen,  2015(33)  Observational 7 N | CABG | 290 (27%)  Groups:  -DAPT held <2d before surgery (n=98) -DAPT held <1d before surgery (n=192) | Indication: Unclear, likely CAD Time:unclear  Age: 65 (10)  Gender: 83% male | Mean Blood Loss at 48 h:  Day -2: 623 mL (IQR 485-913) vs  Day -1: 715 mL, IQR (513-1078 mL)   Plt transfusion:  Day -2: 10% Day -1 : 41% |  |  |  | >2d, ASA+Clop MACE: OR, OR LCI, OR UCI: 0.849, 0.635, 1.135 |  | Not propensity matched, Multiple linear regressions using the logarithm of 48-h blood loss as the dependent variable and the effect of the variable stop day was modelled. | MACCE data is not directly compared between DAPT and other groups. Linear regression using this group on only Median blood loss/Plt transfusion |
| Zhu,  2018(37) Observational 1  N | CABG | 120/180 (66%) Groups:  Treatment group: >1wk DAPT (n=60) Discontinuation: hx DAPT but dc'd >1wk before surgery (n=60) Control: no hx DAPT (n=60) | DAPT indication is CAD + PCI Age: (48.5±3.2) Male: 130/180 | Cont DAPT at least 7 d before surgery   Chest Tube Drainage (total, SD): 1456.8 mL, (680.3 mL) RBC Transfusion: 9.1, (11.2) Plt Transfusion: 0.5, (1.9) | Held DAPT at least 7 d before surgery (discontinue)  Chest Tube Drainage (total, SD): 1254.8 mL (457 mL) RBC transfusion: 6.5 (3.2) Plt Transfusion 0.1 (0.6) |  |  |  |  | N |  |
| Kapoor,  2022(35)  Observational 1 N | CABG | 1200 (100%)  Discontinue >6 d (n=468) D/C 3-5 d (n=621) D/C <2 d (n=111) | "Ages 31-70, no significant diff in age between groups" No gender reported No time since indication reported | < 2 d  RBC transfusion (packed cell volume mL, SD): 34.78, 3.89 CT Drainage (mL, SD): 283.682, 191.915 Re-operation: 10 | 3-5 d RBC transfusion (packed cell volume mL, SD): 35.05, 5.7 CT Drainage (total mL, SD): 216.475, 188.928 Re-operation (Count): 5  6 d RBC transfusion (packed cell volume mL, SD): 28.84, 6.61 CT Drainage (total mL, SD): 333.939, 258.845 Re-operation (Count): 16 |  |  |  |  | No adjustment. Mean/std deviation, ANOVA, chi squared. |  |
| Nardi, 2021(21) Observational 1 N | CABG (on or off pump) | 333 (100%)  Group A: Discontinuing DAPT (ASA + Clopidogrel/Ticagrelor) > 72 hours or 3–4 days (n=159) Group B: Discontinuing Clopidogrel/Ticagrelor (maintaining ASA when possible) 48–72 hours or 2–3 days (n=126) Group C: Discontinuing Clopidogrel/Ticagrelor (maintaining ASA or both agents) < 24 hours or 0–1 days (n=48) | Indication: Coronary artery disease Time: Unspecified Age: A: 67 (8.5), B: 68 (9.8), C: 65 (11.4) Gender (%male): A: 89%, B: 83%, C: 90% | Group C Chest tube drainage (24hrs): 698mL (SD 409) RBC transfusions (total): 0.8u (SD 1.2) Plt transfusions (total): 4 (8.33%) Reoperation for bleeding: 4 (8.33%) | Group A Chest tube drainage (24hrs): 511mL (SD 254) RBC transfusions (total): 0.7u (SD 1.4) Plt transfusions (total): 4 (2.52%) Reoperation for bleeding: 2 (1.25%)  Group B Chest tube drainage (24hrs): 507mL (SD 206) RBC transfusions (total): 1.3u (SD 4.6) Plt transfusions (total): 5 (3.97%) Reoperation for bleeding: 2 (1.59%) |  | Group C Acute MI: 0 All-cause death: 0 | Group A Acute MI: 0 All-cause death: 3 (1.87%)  Group B Acute MI: 0 All-cause death: 1 (0.79%) |  | Variables were compared in an unadjusted analysis. Separate univariate analysis and a logistic regression model were used for additional results not pertinent to the review and so not reported here. |  |
| Tarrant, 2020(19)  Observational 1 Y | Hip surgery (following low energy proximal femur fracture) | 122 (100%)  Compares day of operation after last antiplatelet agent dose (time as continuous variable, 0-9d) (n=122) | Indication: Ischemic heart disease (61%), cerebrovascular disease (31%), peripheral vasular disease (5%), other (3%) Time: Unspecified Age: 83.1 (66-98) Gender: 63% female | Results reported as OR for each day of operative delay after antiplatelet dose RBC transfusions: 1 (0.87-1.15) |  |  | OR for each day of operative delay after antiplatelet dose: All-cause death: 1.32 (1.03-1.68) |  |  | Y: propensity matched on age, sex, Charleston comorbidity index, Nottingham hip fracture score, procedure (arthroplasty: yes/no) | The results were reported as odds ratios of increased risk per day for relevant outcomes as opposed to quantity/ number of events per comparison groups. |
| Cheng, 2020(31)  Observational 1 N | CABG (off pump) | 2012 (100%)  Compares day of DAPT (ASA + Clopidogrel) discontinuation preoperation (time as continuous variable, 0-5d)   0d (n=220)  1d (n=240)  2d (n=360)  3d (n=332)  4d (n=428)  5d (n=432) | Indication: Coronary artery disease Time: Unspecified Age: 61.9 (9.1) Gender: 24.7% female | 0 days Chest tube drainage (mL): 610 (50) RBC transfusions (units): 3.3 (0.4)  Reoperation: 13 (5.9%) BARC 4 major bleeding event within 7d: 64 (29.1%)  1 day Chest tube drainage (mL): 660 (50) RBC transfusions (units): 3 (0.3) Reoperation: 9 (3.6%) BARC 4 major bleeding event within 7d: 59 (24.6%)  2 days Chest tube drainage (mL): 600 (40) RBC transfusions (units): 2.8 (0.9) Reoperation: 17 (4.7%) BARC 4 major bleeding event within 7d: 70 (19.4%) | 3 days Chest tube drainage (mL): 595 (45) RBC transfusions (units): 2.5 (0.7) Reoperation: 6 (1.8%) BARC 4 major bleeding event within 7d: 43 (13%)  4 days Chest tube drainage (mL): 590 (40) RBC transfusions (units): 2.5 (0.5) Reoperation: 10 (2.3%) BARC 4 major bleeding event within 7d: 62 (14.5%)  5 days Chest tube drainage (mL): 560 (35) RBC transfusions (units): 2.6 (0.6) Reoperation: 10 (2.3%) BARC 4 major bleeding event within 7d: 56 (13%) |  |  |  |  | Y: Univariable associations between clinical outcomes and study variables were analyzed using binary logistic regression. | This study included a subgroup analysis of incidence of myocardial ischemia, however did not analyze ischemic outcome by DAPT use, so it was not relevant to this review. |
| Irie, 2019(26)  Observational 1 N | Non-cardiac surgery (emergent, procedure performed within 24hrs of diagnosis) | 133 (100%)  Compares ASA + different P2Y12 inhibitors Groups:  Clopidogrel (n=86)  Ticlodipine (n=37)  Prasugrel (n=10) *All patients received ASA < 5d and P2Y12 < 7d before emergent surgery | Indication: PCI (100%) Time: 982d (0-6433) Age: 74 (38-90) Gender: 73.7% male | Clopidogrel Life threatening or major bleed: 12 (14%)  Ticlodipine Life threatening or major bleed: 3 (8.1%)  Prasugrel Life threatening or major bleed: 3 (30%) |  | Restarting antiplatelet agents earlier than 2d postoperatively Life threatening or major bleed: 11 (8.3%) |  |  |  | Multiple methods: Kaplan-Meier method to describe survival until 180 days after surgery, log-rank test to compare survival between the groups. Multivariable logistic regression. Cox proportional hazard model and estimated hazard ratios (HRs). Covariates were also evaluated for collinearity. | The majority of the results in this study were not useful to our review because the authors' analysis comprised of factors associated with a bleeding and non-bleeding group as opposed to a comparison of DAPT strategies. Additionally, patients received ASA and a P2Y12 inhibitor preop (given urgent nature of surgeries) and medication management consisted of P2Y12 type and restarting agents post op. |
| Vuilliomenet,  2019(36) Observational 1 N | CABG (emergency or urgent) | 262 (78%)  Groups: Time of ticagrelor, prasugrel or clopidogrel d/c before surgery:  <24h (n=101)  24-48h (n=92) 48-72h (n=21) >72h (n=48) | Indication: ACS (100%) Time: ACS within 10 days   Age: Ticagrelor (65.1 (11.0)) Prasugrel (62.8(9.0)) Clopidogrel (67.7(10.9)) Gender:  Ticagrelor (89% male) Prasugrel (85%) Clopidogrel (78%) | d/c ticagrelor <24h Chest tube drainage (cc): 1220 (1197.0) any transfusion (units): 2.5 (17.9) reoperation:  d/c prasugrel <24h Chest tube drainage (cc): 1320 (1934.4) any transfusion (units): 2 (22.5) reoperation:  d/c clopidogrel <24h Chest tube drainage (cc): 1190 (494.3) any transfusion (units): 1 (6.0) reoperation:  d/c ticagrelor 24-48h Chest tube drainage (cc): 1220 (440.0) any transfusion (units): 1 (4.1) reoperation:  d/c prasugrel 24-48h Chest tube drainage (cc): 1050 (742.5) any transfusion (units): 1 (5.2) reoperation:  d/c clopidogrel 24-48h Chest tube drainage (cc): 830 (1319.0) any transfusion (units): 1 (10.6) reoperation: | d/c ticagrelor 48-72h Chest tube drainage (cc): 1100 (260.8) any transfusion (units): 1 (4.5) reoperation:  d/c prasugrel 48-72h Chest tube drainage (cc): 1050 (0) any transfusion (units): 0 (0)  reoperation:  d/c clopidogrel 48-72h Chest tube drainage (cc): 820 (766.7) any transfusion (units): 1 (1.3) reoperation:  d/c ticagrelor >72h Chest tube drainage (cc): 700 (350.7) any transfusion (units): 0 (1.63)  reoperation:  d/c prasugrel >72h Chest tube drainage (cc): 750 (587.8) any transfusion (units): 0 (3.1) reoperation:  d/c clopidogrel >72h Chest tube drainage (cc): 900 (35.5) any transfusion (units): 0 (2.2) reoperation: |  | (note: mortality data not reported by time of discontinuation, only by type of DAPT agent, so not included as DAPT type was not varied) |  |  | multivariable linear regression only for predictors of 24h chest tube output |  |
| Kremke,  2018(20) Observational 3 Y | CABG and/or single valve surgery | 90 (50%)   Groups: Time of ticagrelor d/c before surgery compared to ASA control group:  <72h (n=42) 72-120h (n=48) | Indication: not specified  Time: not specified   Age: DAPT: 68, control: 69 Gender: DAPT: 78% male, control: 80% male | d/c ticagrelor <72h major bleeding: 48% reoperation: 29% | d/c ticagrelor >72h major bleeding: 17% reoperation: 10% |  |  |  |  | Propensity score matching among DAPT group to ASA only control group (by sex, age insulin-dependent DM, COPD, PAD< CNS disease, prior cardiac surgery, critical preop state, unstable angina, reduced LVEF, recent MI, acute surgery, surgery type, ECMO time preop aprotinin use) |  |
| Kacar,  2017(23) Observational 1 N | CABG (within 10d of ACS) | 123 (100%) Groups: Clopidogrel discontinuation before surgery continued (clopidogrel held 1-4 days before surgery) (n=65) discontinued (clopid held 5-10d before surgery) (n=57) | Indication: PCI, 100% Time: Within 10 days Age: Continued: 61.8 (8.1), Discontinued: 60.8 (9.6) Gender: Continued: 68.4% male, Discontinued: 66.7% male | continued (clopidogrel held 1-4 days before surgery) Chest tube drainage (cc, total): 0.65L (in 48hrs) RBC transfusions (total): 0.64L Reoperation: 1 | discontinued (clopid held 5-10d before surgery) (n=57) Chest tube drainage (cc, total): 0.68L (in 48hrs) RBC transfusions (total): 0.47L Reoperation: 1 |  | continued (clopidogrel held 1-4 days before surgery) All-cause death: 0 | discontinued (clopid held 5-10d before surgery) All-cause death: 0 |  | no multivariable models for outcomes of interest reported |  |
| Altun,  2017(38)  RCT 1 N | CABG | Pre-Op DAPT: 54, 100% TnX-A (n = 18) TnX-A+Des (n = 16) Des (n = 10) Control (n = 10) | ACS, 100%  Male TnX-A 84%, 65.8 ± 6.1 TnX-A+Des 88%, 65.6 ± 11.3  Des 90%, 66.4 ± 9.3 Control 90%, 57.9 ± 14.6 | DAPT in all groups TnX-A  Total Blood Loss (chest tube drainage mL, SD): 535, 116.8  RBC transfusion (erythro suspe mL): 125, 128.6 Platelet sus (mL): 0, 0  TnX-A + Des  Total Blood Loss (chest tube drainage mL, SD): 574, 75.5 RBC transfusion (erythro suspe mL): 93.7, 125 Platelet sus (mL): 0, 0  Des alone  Total Blood Loss (chest tube drainage mL, SD): 1430, 257.6 RBC transfusion (erythro suspe mL): 675, 237.1 Platelet sus (mL): 0, 0  Control (no drug) Total Blood Loss (chest tube drainage mL, SD): 1767.5, 293.2.  RBC transfusion (erythro suspe mL): 900, 268.7 Platelet sus (mL): 120, 209.7 |  |  |  |  |  | N |  |
| Doğan, 2017(29)  Observational  1 N | Renal transplant | 106 (100%)  Compares groups with variable timing since stent placement interruption Groups:   DES-Early 3mo from DES implantation (n=41)  DES-Late- 3-12mo from DES implantation  BMS- at least 1mo from BMS implantation *Interruption defined as holding ASA and Clopidogrel 5-7d before transplant | Indication: Stable angina, unstable angina, or NSTEMI Timing: Variable per group Age: BMS: 58.17 (5.4), DES-Early: 54.55 (6.6), DES-Late: 56.63 (6.9) Gender (%male): BMS: 75%, DES-Early: 65.9%, DES-Late: 65.9% |  |  |  |  | DES-Early MACE: 2 (4.9%) Acute MI: 1 (2.4%) CV Death: 0 All-Cause Death: 1 (2.4%)  DES-Late MACE: 3 (7.3%)  Acute MI: 2 (2.9%) CV Death: 0 All-Cause Death: 1 (2.4%)  BMS MACE: 2 (8.3%) Acute MI: 1 (4.2% CV Death: 1 (4.2%) All-Cause Death: 2 (8.3%) |  | No adjustment | All patients had DAPT held 5-7 days prior to surgery. The timing since DAPT indication was varied. |
| Della Corte,  2017(22) Observational 1 N | CABG | 226 (100%) Groups: time of d/c clopidogrel or ticagrelor 0-3 days (n=34) >3 days (n=192) | Indication: not specified  Time: not specified  Age: 63 (9) Gender: 80.5% male | d/c clopidogrel 0-3d  Post-op blood loss: 700 (205.9)   d/c ticagrelor 0-3d  Post-op blood loss: 800 (577.8) | d/c clopidogrel >4d  Post-op blood loss: 625 (264.4)  d/c ticagrelor >4d  Post-op blood loss: 560 (270.4) |  |  |  |  | multivariable logistic regression | Other outcomes (including transfusions, reexploration) only compared clopidogrel versus ticagrelor groups instead of comparing time to agent discontinuation so are not reported here |
| De Servi, 2015(32) Observational  3 N | Cardiac/ Vascular/ Uro/ Abd/ Thoracic/ Ortho/ Other | Pre-Op DAPT: (100%)  Bridge P2Y12 inhibitor with i.v. tirofiban.(n=87)  Control (continue or d/c P2Y12 inhibitor without Bridge) (n=227) | DAPT Indication : PCI 6-12 months, 100% Time (days): Bridge: 104 [5–365]; control: 105 [0–360] Age: Bridge: 67.4 [25–83]; control: 69.2 [41–90] Gender: Bridge: 64 (73.6% male), Control: 180 (79.3% male) |  |  | Bridge  TIMI major bleeding 5 (5.7%)  Any transfusion 22 (25.9%)  Control (no bridge)   TIMI major bleeding: 36 (15.8%)   Any transfusion: 76 (33.5%) |  |  | Bridge  MACCE: 2 (2.3%) Stroke: 0 Death: 0 MI: 2 (2.34%)   Control (no bridge)  MACCE: 17 (7.5%) Stroke: 0 Death: 6 (2.6%) MI: 12 (5.3%) | multivariable logistic regression (only used for net adverse cardiac events which was not abstracted for consistency across studies)  Nearest-neighbor matching, the bridge therapy did not show a statistically significant effect on overall MACE (4% lower in the treated sample, p = 0.199). |  |
| Hansson,  2015  Observational 8 Y | CABG | Pre-Op DAPT  Ticagrelor+ASA n = 1266 (56.4%)  Clopidogrel+ASA n = 978, 43.5% | DAPT indication- ACS Time: Unspecified Clop+ASA Age: 68.4 +/- 9.5 Gender: 775/978 (79.2%) Tica + ASA Age: 67.8 +/- 9.4 Gender: 995/1266 (78.5%) | d/c clopidogrel 0-24 hours  Blood Loss (mL, SD): 663, 627 RBC Transfusion (units, SD): 4.9, 6.8 Platelet Transfusion (units, SD): 1.5, 2.3   d/c clopidogrel 24-48 hours  Post op blood loss (mL, SD): 714, 462 RBC Transfusion (units, SD): 3.4, 4.5 Platelet Transfusion (units, SD): 0.94, 1.5  d/c ticagrelor 0-24 hours  Blood Loss (mL): 813, 478 RBC Transfusion (units, SD): 6.9, 9.8 Platelet Transfusion (units, SD): 3.2, 3.7   d/c ticagrelor 24-48 hours  Post op blood loss (mL, SD): 641, 337 RBC Transfusion (units, SD): 4.4, 5.7 Platelet Transfusion (units, SD):1.6, 2.2 | d/c clopidogrel 48-72 hours   Post op blood loss (mL, SD): 659, 313 RBC Transfusion (units, SD): 2.8, 3.5 Platelet Transfusion (units, SD): 0.79,1.4  d/c clopidogrel 72-96 hours  Post op blood loss (mL, SD): 682, 462 RBC Transfusion (units, SD): 3, 5.3 Platelet Transfusion (units, SD): 0.68, 1.4  d/c clopidogrel 96-120 hours  Post op blood loss (mL, SD): 701, 454 RBC Transfusion (units, SD): 2.3, 2.9 Platelet Transfusion (units, SD): 0.51, 1  d/c clopidogrel >120 hours  Post op blood loss (mL, SD): 555, 313 RBC Transfusion (units, SD): 1.7, 3 Platelet Transfusion (units, SD): 0.25, 0.84  d/c ticagrelor 48-72 hours  Post op blood loss (mL, SD): 709, 707 RBC Transfusion (units, SD): 4, 9.9 Platelet Transfusion (units, SD): 1.8, 3.7  d/c ticagrelor 72-96 hours  Post op blood loss (mL, SD): 630, 541 RBC Transfusion (units, SD): 1.7, 3.2 Platelet Transfusion (units), SD: 0.44, 0.81  d/c ticagrelor 96-120 hours  Post op blood loss (mL, SD): 550, 296 RBC Transfusion (units), SD: 1.3, 2.1 Platelet Transfusion (units, SD): 0.32, 0.9  d/c ticagrelor >120 hours  Post op blood loss (mL, SD): 534, 363 RBC Transfusion (units, SD): 1.6, 3.2 Platelet Transfusion (units, SD): 0.24, 0.95 |  |  |  |  | Y: Multivariable logistic regression |  |
| Cao, 2021(27) Observational 1 N | Non-cardiac surgery | 747 (81.7%)  Groups:   DAPT interruption (any kind) (n=297)  No DAPT interruption (n=312)  ASA + P2Y12 interruption (n=128)  Only P2Y12 interruption (n=152) | Indication: PCI, 100% Time: <1yr since PCI Age: Not specified Gender: 67.6% male | No DAPT interruption  Bleeding (defined as requiring >2u RBC transfusion): 26 (8.3%) | DAPT interruption (any kind) Bleeding (defined as requiring >2u RBC transfusion): 40 (13.5%)  ASA + P2Y12 interruption Bleeding (defined as requiring >2u RBC transfusion): 25 (19.5%)  Only P2Y12 interruption Bleeding (defined as requiring >2u RBC transfusion): 14 (9.3%) |  | No DAPT interruption: MACE: 11 (3.5%) Acute MI: 3 (1%) All-cause death: 8 (2.6%) | DAPT interruption (any kind) MACE: 8 (2.7%) Acute MI: 2 (0.7%) All-cause death: 6 (2%)  ASA + P2Y12 interruption MACE: 5 (3.9%) Acute MI: 1 (0.8%) All-cause death: 4 (3.1%)  Only P2Y12 interruption MACE: 2 (1.3%) Acute MI: 1 (0.7%) All-cause death: 1 (0.7%) |  | Y: Multivariable logistic regression. Variables for risk-adjustment: age, sex, urgent/emergent surgery, risk category (low, intermediate or high), and ASA-PS class. |  |
| Shahid,  2021(24) Observational 1 N | CABG | 192 (100%)  Group A: d/c clopidogrel < 48h (n=102)  Group B: d/c clopidogrel 48-120h before surgery (n=89) | ACS, 100%  Male TnX-A group 84%, 65.8 ± 6.1 TnX-A+Des 88%, 65.6 ± 11.3  Des 90%, 66.4 ± 9.3 Control 90%, 57.9 ± 14.6 | Group A: d/c clopidogrel < 48h Chest tube drainage (cc, total): 602.25 (200) Any transfusion: 33 (32%)  Reoperation: 3 (2.9%) | Group B: d/c clopidogrel 48-120h Chest tube drainage (cc, total): 609.87 (200) Any transfusion 25 (28.1%)  Reoperation: 1 (1.1%) |  | Group A: d/c clopidogrel < 48h All-cause death: 7 (6.8%) | Group B: d/c clopidogrel 48-120h All-cause death: 2 (2.2%) |  | N, all data are unadjusted |  |
| Kim, 2020(28) Observational  9 N | Non-cardiac surgery | Total n=3582    Continue DAPT: n=984, (27.4%)  Discontinue APT n=1750, (49%) | Indication for DAPT: PCI    Time since indication, mean months (SD): not specified  Age, years mean (SD): 69 (61-75)  Gender, %male: 1282 (70) |  | HR: Incidence Major bleeding d/c 1-3 days  OR, OR LIC, OR UCI: 1.54, 0.85, 2.8  d/c 4-8 days OR, OR LIC, OR UCI: 0.89, 0.55, 1.44  d/c: at least 9 days  OR, OR LIC, OR UCI: 1.5, 0.76, 2.97 |  | Continue DAPT MACE: 47 (4.8%) | Discontinue DAPT MACE (events): 36 (4.5%) |  | multivariate logistic regression model (Note: in an additional model looking at holding >8 days, they reported higher adjusted MACE compared to <8d (adjusted HR, 3.38; 95% CI, 1.36–8.38; P=0.009)) |  |
| Heidari  2016(25) Observational  1 N | CABG | 100 (66%)  Group A: DAPT continued, urgent CABG, experienced surgeon (n=50) Group C: DAPT held > 5d, elective CABG, experienced surgeon (n=50) (*Group B not relevant - does not vary DAPT) | Indication: ACS Time: not specified  Age, years mean (SD): A: 59.5 (9.70), C: 57.9 (8.70)  Gender, %male: A: 72, C: 66 | DAPT continued (Group A) RBC transfusions (units): 0.78 (1.14) Intraop blood loss volume (cc): 987.9 (443) Reoperation: 0 | DAPT held >5d (Group C)  RBC transfusions (units): 3.14 (1.9)  Intraop blood loss volume (cc): 973 (537.5)  Reoperation: 0 |  | DAPT continued (Group A) All-cause death: 0 (0%) [in-hospital] | DAPT held >5d (Group C) All-cause death: 0(0%) [in-hospital] |  | N, all data are unadjusted. (Group B was urgent CABG with DAPT continuation with empiric transfusions given and inexperienced surgeons. Given that the DAPT management did not vary, we determined A and C groups were the comparison of interest, and C was not randomized) |  |

*Notes***.** Mean (SD) unless otherwise specified; median [IQR].

*Abbreviations*. ACS=acute coronary syndrome; CV=cardiovascular; d/c=discontinue; MACE=major adverse cardiovascular events; MACCE=all-cause death, myocardial infarction, definite stent thrombosis and stroke; MALE=major adverse limb; MI=myocardial infarction; ns=not significant; OR=odds ratio.

Appendix E. Excluded Studies

No Relevant Outcome Data Presented for the Patients that were on Preoperative DAPT Comparing at Least 2 Perioperative Strategies, *N* = 38

1. Altun, G., et al., Emergency coronary bypass surgery in patients under the influence of dualantiplatelet therapy: effects of tranexamic acid and desmopressin acetate. Turk J Med Sci, 2017. 47(6).

2. Amour, J., et al., Prospective observational study of the effect of dual antiplatelet therapy with tranexamic acid treatment on platelet function and bleeding after cardiac surgery. Br J Anaesth, 2016. 117(6): p. 749-757.

3. Awada, H., et al., Pocket related complications following cardiac electronic device implantation in patients receiving anticoagulation and/or dual antiplatelet therapy: prospective evaluation of different preventive strategies. J Interv Card Electrophysiol, 2019. 54(3): p. 247-255.

4. Benkö, T., et al., One-year Allograft and Patient Survival in Renal Transplant Recipients Receiving Antiplatelet Therapy at the Time of Transplantation. Int J Organ Transplant Med, 2018. 9(1): p. 10-19.

5. Charif, F., et al., Dual antiplatelet therapy up to the time of non-elective coronary artery bypass grafting with prophylactic platelet transfusion: is it safe? J Cardiothorac Surg, 2019. 14(1): p. 202.

6. Chemtob, R.A., et al., Outcome After Surgery for Acute Aortic Dissection: Influence of Preoperative Antiplatelet Therapy on Prognosis. J Cardiothorac Vasc Anesth, 2017. 31(2): p. 569-574.

7. Christersson, C., et al., Comparison of warfarin versus antiplatelet therapy after surgical bioprosthetic aortic valve replacement. Heart, 2020. 106(11): p. 838-844.

8. Cui, R.B.J., K.S. Ng, and C.J. Young, Complications Arising From Perioperative Anticoagulant/Antiplatelet Therapy in Major Colorectal and Abdominal Wall Surgery. Dis Colon Rectum, 2018. 61(11): p. 1306-1315.

9. Dai, Y., et al., Dual antiplatelet therapy increases pocket hematoma complications in Chinese patients with pacemaker implantation. Journal of geriatric cardiology, 2015. 12(4): p. 383‐387.

10. Deharo, J.C., et al., Perioperative management of antithrombotic treatment during implantation or revision of cardiac implantable electronic devices: the European Snapshot Survey on Procedural Routines for Electronic Device Implantation (ESS-PREDI). Europace, 2016. 18(5): p. 778-84.

11. Egholm, G., et al., Dual anti-platelet therapy after coronary drug-eluting stent implantation and surgery-associated major adverse events. Thromb Haemost, 2016. 116(1): p. 172-80.

12. Guo, J., et al., Effects of Sarpogrelate Combined with Aspirin in Patients Undergoing Carotid Endarterectomy in China: A Single-Center Retrospective Study. Ann Vasc Surg, 2016. 35: p. 183-8.

13. Hansson, E.C., et al., Preoperative dual antiplatelet therapy increases bleeding and transfusions but not mortality in acute aortic dissection type A repair. Eur J Cardiothorac Surg, 2019. 56(1): p. 182-188.

14. Howell, S.J., et al., Prospective observational cohort study of the association between antiplatelet therapy, bleeding and thrombosis in patients with coronary stents undergoing noncardiac surgery. Br J Anaesth, 2019. 122(2): p. 170-179.

15. Hudson, J.S., et al., Hemorrhage associated with ventriculoperitoneal shunt placement in aneurysmal subarachnoid hemorrhage patients on a regimen of dual antiplatelet therapy: a retrospective analysis. J Neurosurg, 2018. 129(4): p. 916-921.

16. Hussain, A., et al., Is the use of dual antiplatelet therapy following urgent and emergency coronary artery bypass surgery associated with increased risk of cardiac tamponade? J Clin Transl Res, 2021. 7(2): p. 229-233.

17. Jones, D.W., et al., Dual antiplatelet therapy reduces stroke but increases bleeding at the time of carotid endarterectomy. J Vasc Surg, 2016. 63(5): p. 1262-1270.e3.

18. Kawamoto, Y., et al., Effect of antithrombic therapy on bleeding complications in patients receiving emergency cholecystectomy for acute cholecystitis. Journal of Hepato-Biliary-Pancreatic Sciences, 2018. 25(11): p. 518-526.

19. Kyuchukov, D., I. Zheleva-Kyuchukova, and G. Nachev, Antithrombotic regimens in patients after coronary artery bypass grafting and coronary endarterectomy. Pharmacia, 2020. 67(3): p. 115-120.

20. Lin, S.Y., et al., The Safety of Continuing Antiplatelet Medication Among Elderly Patients Undergoing Urgent Hip Fracture Surgery. Orthopedics, 2019. 42(5): p. 268-274.

21. Mishu, M.D., et al., Should Antiplatelet Therapy Be Withheld Perioperatively? The First Study Examining Outcomes in Patients Receiving Dual Antiplatelet Therapy in the Lower Extremity Free Flap Population. Plast Reconstr Surg, 2022. 149(1): p. 95e-103e.

22. Nagashima, Z., et al., Impact of preoperative dual antiplatelet therapy on bleeding complications in patients with acute coronary syndromes who undergo urgent coronary artery bypass grafting. J Cardiol, 2017. 69(1): p. 156-161.

23. Oh, T.K., C. Im, and I.A. Song, Antiplatelet Therapy in Patients Without a Coronary Stent and Mortality After Noncardiac Surgery. Journal of Surgical Research, 2020. 256: p. 61-69.

24. Ohya, H., et al., Comparison of the continuation and discontinuation of perioperative antiplatelet therapy in laparoscopic surgery for colorectal cancer: A retrospective, multicenter, observational study (YCOG 1603). Annals of Gastroenterological Surgery, 2021. 5(1): p. 67-74.

25. Park, S.K., et al., Risk of non-cardiac surgery after percutaneous coronary intervention with drug-eluting stents. Sci Rep, 2017. 7(1): p. 16393.

26. Plicner, D., et al., Preoperative platelet aggregation predicts perioperative blood loss and rethoracotomy for bleeding in patients receiving dual antiplatelet treatment prior to coronary surgery. Thrombosis research, 2015. 136(3): p. 519‐525.

27. Rossini, R., et al., Antiplatelet therapy and outcome in patients undergoing surgery following coronary stenting: Results of the surgery after stenting registry. Catheter Cardiovasc Interv, 2017. 89(1): p. E13-e25.

28. Sadeghi, R., et al., Dual antiplatelet therapy before coronary artery bypass grafting in patients with myocardial infarction: a prospective cohort study. BMC Surg, 2021. 21(1): p. 449.

29. Schaefer, A., et al., Preoperative Ticagrelor administration leads to a higher risk of bleeding during and after coronary bypass surgery in a case-matched analysis. Interact Cardiovasc Thorac Surg, 2016. 22(2): p. 136-40.

30. Schlachtenberger, G., et al., Major Bleeding after Surgical Revascularization with Dual Antiplatelet Therapy. Thorac Cardiovasc Surg, 2020. 68(8): p. 714-722.

31. Smith, B.B., et al., Cardiac Risk of Noncardiac Surgery After Percutaneous Coronary Intervention With Second-Generation Drug-Eluting Stents. Anesth Analg, 2019. 128(4): p. 621-628.

32. Straus, S., et al., A Difference in Bleeding and Use of Blood and Blood Products in Patients who Were Preoperatively on Aspirin or Dual Antiplatelet Therapy Before Coronary Artery Bypass Grafting. Med Arch, 2018. 72(1): p. 31-35.

33. Sun, J., et al., Safety and feasibility study of holmium laser enucleation of the prostate (HOLEP) on patients receiving dual antiplatelet therapy (DAPT). World J Urol, 2018. 36(2): p. 271-276.

34. Tianchetsada, N. and A. Suwanagool, Antithrombotic management and device-related bleeding complications in patients undergoing cardiac implantable electronic device implantations: A single-center study. Journal of the Medical Association of Thailand, 2018. 101(1): p. 33-39.

35. Ueoka, K., et al., The influence of pre-operative antiplatelet and anticoagulant agents on the outcomes in elderly patients undergoing early surgery for hip fracture. J Orthop Sci, 2019. 24(5): p. 830-835.

36. Xiao, F.C., et al., Does preoperative dual antiplatelet therapy affect bleeding and mortality after total arch repair for acute type A dissection? Interact Cardiovasc Thorac Surg, 2022. 34(1): p. 120-127.

37. Yoshimoto, M., et al., Emergent cholecystectomy in patients on antithrombotic therapy. Sci Rep, 2020. 10(1): p. 10122.

38. Yoshimoto, Y., et al., Optimal use of antiplatelet agents, especially aspirin, in the perioperative management of colorectal cancer patients undergoing laparoscopic colorectal resection. World Journal of Surgical Oncology, 2019. 17(1).

Endovascular, *N =* 3

1. Chinai, N., et al., Single versus dual antiplatelet therapy following peripheral arterial endovascular intervention for chronic limb threatening ischaemia: Retrospective cohort study. PLoS One, 2020. 15(6): p. e0234271.

2. Ghamraoui, A.K., et al., Clopidogrel versus ticagrelor for antiplatelet therapy in transcarotid artery revascularization (TCAR) in the Society for Vascular Surgery Vascular Quality Initiative. J Vasc Surg, 2021.

3. Kronlage, M., et al., Anticoagulation in addition to dual antiplatelet therapy has no impact on long-term follow-up after endovascular treatment of (sub)acute lower limb ischemia. Vasa, 2019. 48(4): p. 321-329.

DAPT Interruption Not Specified, *N =* 1

1. Humenberger, M., M. Stockinger, S. Kettner, J. Siller-Matula and S. Hajdu (2019). "Impact of Antiplatelet Therapies on Patients Outcome in Osteosynthetic Surgery of Proximal Femoral Fractures." J Clin Med 8(12).

Does Not Specify Dual Antiplatelet, *N =* 1

1. Hong, S. J., M. J. Kim, J. S. Kim, E. H. Kim, J. Lee, C. M. Ahn, B. K. Kim, Y. G. Ko, D. Choi, M. K. Hong and Y. Jang (2019). "Effect of Perioperative Antiplatelet Therapy on Outcomes in Patients With Drug-Eluting Stents Undergoing Elective Noncardiac Surgery." American Journal of Cardiology **123**(9): 1414-1421.

No Outcome of Interest, *N =1*

1. Kim, C., J. S. Kim, H. Kim, S. G. Ahn, S. Cho, O. H. Lee, J. K. Park, S. Shin, J. Y. Moon, H. Won, Y. Suh, J. R. Cho, Y. H. Cho, S. J. Oh, B. K. Lee, S. J. Hong, D. H. Shin, C. M. Ahn, B. K. Kim, Y. G. Ko, D. Choi, M. K. Hong and Y. Jang (2021). "Consensus decision-making for the management of antiplatelet therapy before non-cardiac surgery in patients who underwent percutaneous coronary intervention with second-generation drug-eluting stents: A cohort study." Journal of the American Heart Association **10**(8).

Not at Least 2 Comparison Groups of Patients on DAPT, *N = 1*

1. Hu, S. B., Y. Hai, J. F. Tang, T. Liu, B. X. Liang and B. Q. Xue (2019). "Risk of bleeding in patients with continued dual antiplatelet therapy during orthopedic surgery." Chin Med J (Engl) **132**(8): 943-947.

Single Arm with Bridging, *N = 1*

1. Dargham, B. B., A. Baskar, I. Tejani, Z. Cui, S. Chauhan, J. Sum-Ping, R. A. Weideman and S. Banerjee (2019). "Intravenous Antiplatelet Therapy Bridging in Patients Undergoing Cardiac or Non-Cardiac Surgery Following Percutaneous Coronary Intervention." Cardiovasc Revasc Med 20(9): 805-811.

TAVR, *N =1*

1. Hioki, H., Y. Watanabe, K. Kozuma, Y. Nara, H. Kawashima, A. Kataoka, M. Yamamoto, K. Takagi, M. Araki, N. Tada, S. Shirai, F. Yamanaka and K. Hayashida (2017). "Pre-procedural dual antiplatelet therapy in patients undergoing transcatheter aortic valve implantation increases risk of bleeding." Heart 103(5): 361-367.

Unavailable, *N = 1*

1. Zhang, J., F. Huang, J. Yang, Q. Wu, Y. Liu, Y. Zhou, Y. Zou and E. Zhu (2015). "Impact of preoperative dual antiplatelet therapy on perioperative bleeding in patients undergoing off-pump coronary artery bypass grafting." National medical journal of china **95**(24): 1934‐1937
